# Promoting community participation in priority setting in district health systems: experiences from Mbarali district, Tanzania

**DOI:** 10.3402/gha.v6i0.22669

**Published:** 2013-11-25

**Authors:** Peter Kamuzora, Stephen Maluka, Benedict Ndawi, Jens Byskov, Anna-Karin Hurtig

**Affiliations:** 1 Institute of Development Studies, University of Dar es Salaam, Dar es Salaam, Tanzania; 2 Primary Health Care Institute (PHCI), Iringa, Tanzania; 3 DBL-Centre for Health Research and Development, Faculty of Health and Medical Sciences, University of Copenhagen, Frederiksberg, Denmark; 4 Umeå International School of Public Health (UISPH), Umeå University, Umeå, Sweden

**Keywords:** community participation, priority setting, district health systems, Tanzania

## Abstract

**Background:**

Community participation in priority setting in health systems has gained importance all over the world, particularly in resource-poor settings where governments have often failed to provide adequate public-sector services for their citizens. Incorporation of public views into priority setting is perceived as a means to restore trust, improve accountability, and secure cost-effective priorities within healthcare. However, few studies have reported empirical experiences of involving communities in priority setting in developing countries. The aim of this article is to provide the experience of implementing community participation and the challenges of promoting it in the context of resource-poor settings, weak organizations, and fragile democratic institutions.

**Design:**

Key informant interviews were conducted with the Council Health Management Team (CHMT), community representatives, namely women, youth, elderly, disabled, and people living with HIV/AIDS, and other stakeholders who participated in the preparation of the district annual budget and health plans. Additionally, minutes from the Action Research Team and planning and priority-setting meeting reports were analyzed.

**Results:**

A number of benefits were reported: better identification of community needs and priorities, increased knowledge of the community representatives about priority setting, increased transparency and accountability, promoted trust among health systems and communities, and perceived improved quality and accessibility of health services. However, lack of funds to support the work of the selected community representatives, limited time for deliberations, short notice for the meetings, and lack of feedback on the approved priorities constrained the performance of the community representatives. Furthermore, the findings show the importance of external facilitation and support in enabling health professionals and community representatives to arrive at effective working arrangement.

**Conclusion:**

Community participation in priority setting in developing countries, characterized by weak democratic institutions and low public awareness, requires effective mobilization of both communities and health systems. In addition, this study confirms that community participation is an important element in strengthening health systems.

Community participation in priority setting in health systems has gained importance all over the world, particularly in resource-poor settings where governments have often failed to provide adequate public-sector services for their citizens. Incorporation of public views into priority setting is perceived as a means to restore trust, improve quality of healthcare and health outcomes, better accountability, and more efficient use of resources (1–3). In addition, the participation of those affected by policy decisions in planning and priority setting is a key contribution as it will include their distinct personal experience and non-medical understanding of issues which in turn can lead to asking questions that health workers may not have considered (4).

Community participation has been a central theme in health-related discussions for many years. It is present in the World Health Organization (WHO) constitution, confirmed in the Alma-Ata Declaration. The fourth principle of the Declaration stated that ‘The people have the right and duty to participate individually and collectively in the planning and implementation of their health care’ (1). Other major WHO reports, such as the Ottawa Charter for Health Promotion (5) and Jakarta Declaration (6) also emphasized the importance of community participation as a key principle for successful health system strengthening.

While the concept of participation is not new to the health sector, it is a contentious term that can mean everything and nothing (7). The meanings can vary from allowing community representatives a seat at the table where policy decisions are made; to people being involved in agenda setting, analyzing problems and participating in decision making; to a process of democratization whereby governments become more accountable and responsive to the needs of the disenfranchised; to a cost-sharing exercise contributing toward sustainable programs (8).

In this article, the term community participation is understood as the collective involvement of local people in assessing and prioritizing their needs and organizing strategies to meet those needs (9).

Globally, there is increasing evidence on the formation of health committees or boards involving community representatives (10). However, actual implementation of decentralization strategies to ensure the full potential of community participation still remains limited in a number of developing countries (10). In the context of Tanzania, community participation has emerged as an important dimension in healthcare planning and decision making within decentralized district healthcare systems across the country. The central government in Tanzania is increasingly pushing responsibility for planning and delivery of health services closer to communities, and civil society and citizens are being asked to play a greater participatory role in these processes.

However, while community participation has gained currency over the past two decades or so, studies indicate that despite existence of health committees and boards, community views are rarely taken into consideration in district decision-making processes (11–14). Similarly, the baseline study conducted as part of the Response to Accountable Priority Setting for Trust in health system (REACT) project intervention reported in this study revealed that while user committees and boards had been established in the district, they had little impact on the planning and priority-setting process (15–17). Consequently, district health plans were products of a few members of the Council Health Management Team (CHMT), with private partners and community bodies at best operating as a rubber stamp for decisions taken without their input. Furthermore, it was reported that some members of the boards and committees were not active, some not replaced, and often they did not know what was expected of them (15).

Nevertheless, while studies on community participation in healthcare exist, there is scant knowledge on how to promote community participation in planning and priority setting in resource-constrained settings. The aim of this article is to provide the experience of implementing community participation and the challenges of promoting it in the context of resource-poor settings, weak organizations, and fragile democratic institutions.

## Methodology

### Study context

The study was conducted in Mbarali district in the Mbeya region of Tanzania. Mbarali district was selected by the REACT project, as it was a typical rural district in Tanzania. Table 1 summarizes key demographic and health indicators of the study district.

**Table 1:** Key demographic and health indicators of the study district

Indicator	National	Mbarali district
Total population	33,461,849	300,517
Growth rate	2.9%	2.8%
Fertility rates	5.4	4
Children <1 year	4.0%	4%
Children <5 years	21%	20%
Women: 15–49	18%	20%
Maternal mortality	454/100,000	247/100,000
Under-five mortality	81/1,000	104/1,000

*Source:* Tanzania Census report, 2012 (18), and Demographic and Health Survey, 2010 (19).

Similar to other districts in Tanzania, the structure of the health system in Mbarali district has been decentralized. At the district level, the CHMT was formed with the remit of planning and budgeting for activities needed to manage, control, coordinate, and support all health services in the district on a year-to-year basis.

To ensure that the district health plans are in line with national health strategies, in 2000 the Ministry of Health developed the National Package of Essential Health Interventions as a way of ensuring that the highest priority services are fully supported. Based on this national framework, all districts produce an annual Comprehensive Council Health Plan (CCHP), which incorporates all activities of the District Health Services, and all sources of funding at the council level (government funds, locally generated funds, local donor funds, etc.).

The CCHP is approved by the Council Health Services Board (CHSB), which consists of community representatives, officers from other departments, and representatives from the private sector. The final plan is approved at the Full Council Meeting. The Regional Secretariat (Regional Health Management Team) reviews and approves the CCHP and forwards it to national level. The Prime Minister's Office-Regional Administration and Local Government (PMO-RALG), together with the Ministry of Health and Social Welfare (MOHSW), assesses the CCHPs and must give its final approval before funds can be disbursed to the local government authorities. Table 2 summarizes structures of the district health systems and their roles and functions.

**Table 2:** Composition, functions, and roles of the health facility committees and boards in Tanzania

Composition		Functions and roles
1.	Regional Health Management Team (RHMT) consists of 8 core members: the Regional Medical Officer (chairperson), Regional Nursing Officer, Regional Laboratory Technician, Regional Health Officer, Regional Pharmacist, Regional Dental Officer, District Regional Health Secretary (secretary), and Regional Social Welfare Officer.	Technical advisors to CHMT Prepare regional annual health plans Discuss and approval health plans, budget and reports from the CHMT
2.	The Council Health Service Board (CHSB) consists of 11 members: 4 non-vote members (DMO, RHMT, Planning Officer and representative from the hospital) and 7 vote members including 4 elected community members of whom at least 2 should be female. Other 3 members are 2 representatives from private-service providers and chairperson of social services committee of the council Qualification is secondary education and above and of age 25–70 years	Identify, mobilize and solicit financial resources Ensure delivery of healthcare services Discuss and approval health plans, budgets, and reports from the CHMT Support CHMT in managing and administering health resources Promote community involvement through sensitization Liaise with other partners with similar interests.
3.	Council Health Management Team (CHMT) consists of 8 core members: the District Medical Officer (chairperson), District Nursing Officer, District Laboratory Technician, District Health Officer, District Pharmacist, District Dental Officer, District Health Secretary (secretary), and District Social Welfare Officer.	Prepare district annual health plans Ensure implementation of health activities by hospitals, health centers, dispensaries, and communities Ensure that data are used by health workers to plan and implement interventions Monitor and evaluate implementation of health activities in the district.
4.	The Hospital Governing Committee is established at the hospital and consists of 10 members: seven vote members (2 service users, 1 from health center committee, 1 DC, 1 from voluntary facility and 1 from NGO); and 3 non-vote members (medical officer in-charge, office of the DMO, and a representative from the CHSB).	Oversee management of resources at the hospital Discuss and pass proposals and budgets for the hospital and submit to the council through CHSB Discuss implementation report from hospital management team Inform communities on hospital plans and its implementation.
5.	Health Centre Committees are composed of 8 members: 6 vote members (3 service users, 1 from dispensary committee, 2 from private providers); and 2 non-vote members (head of the facility and 1 from WDC).	Discuss implementation reports prepared by the Health Centre Management Team Identify and solicit financial resources for running Health Centre Services Advice and recommend to the CHSB on human resources development in terms of recruitment, training, deployment, and motivation
6.	Dispensary Committees are composed of 8 members: 5 vote members (3 service users, 2 from private providers); and 3 non-vote members (1 from WDC, 1 from village government and 1 head of the dispensary).	Discuss and pass dispensary plans and budgets; identify and solicit funds; Assist Dispensary Management team in planning and managing community-based initiatives Ensure delivery of appropriate services

*Source*: From Ref. (20).

### The REACT intervention in Tanzania

In 2006, researchers from Tanzanian institutions (the Primary Health Care Institute, the Institute of Development Studies, the University of Dar es Salaam, and the National Institute for Medical Research, in collaboration with research institutions from Europe) teamed with decision makers and launched the project known as Response to Accountable Priority Setting for Trust in health systems (REACT) in Kenya, Zambia, and in Tanzania where the study was carried out. REACT was a 5-year project aimed at testing the application and effects of Accountability for Reasonableness (AFR) approach to priority setting in resource-constrained settings. AFR is a comprehensive framework which provides structure for stakeholders to establish priorities for their specific contexts, while taking into account limited resources and regulatory conditions. The REACT project aimed at implementing the four conditions of the AFR framework (see Table 3).

**Table 3:** Four conditions of the AFR

Relevance	The rationales for priority-setting decisions must be based on evidence, reasons, and principles that providers and users can agree are relevant to meeting healthcare needs fairly under reasonable resource constraints.
Publicity	Priority-setting decisions, and the grounds for making them, must be publicly accessible through various forms of active communication outreach. Transparency should open decisions and their rationales to scrutiny by all those affected by them, not just the members of the decision-making group.
Appeals and revision	There must be a mechanism for challenge, including the processes for revising decisions and policies in response to new evidence, individual considerations, and as lessons are learnt from experience.
Enforcement/leadership and public regulation	Local systems and leaders must ensure that the above three conditions are met.

The implementation of the REACT project in Mbarali district was through participatory action research involving the CHMT members and an Action Research Team (ART). The action research process involved: (i) the study of existing priority-setting practices and structures, (ii) provision of training and coaching to members of the CHMT and other decision makers to generate knowledge necessary to transform the organization's priority-setting practices, and (iii) action toward the development and implementation of improvement strategies in a continuous process to address gaps in AFR conditions.

A preliminary phase of the implementation of the AFR framework began in 2006, involving gathering baseline data, consultation, and planning. The full application of AFR began in 2008. The ART comprised four members of the CHMT and two researchers from research and academic institutions. The two researchers were from the Primary Health Care Institute in Iringa and the Institute of Development Studies, University of Dar es Salaam. The role of the CHMT was to introduce the application of AFR conditions during the annual planning and priority setting at the district level, and in day-to-day decision-making processes that concern prioritization within tight resource limits.

However, while different stakeholders in the district were sensitized about the AFR framework, the researchers predominantly worked with the existing health-related structures (the CHMT members) that are responsible for preparing district health plans and budgets. The intention was that once they ‘get it’, the CHMT members would then improve the degree of community engagement in the district annual planning and budgeting.

The ART team conducted meetings once every 2 months to discuss and review the implementation of AFR in the district. Additionally, the researchers held meetings with the CHMT every 6 months to discuss and review the application of AFR conditions in the district. Furthermore, all collaborating research institutions of the REACT project held annual workshops to review and discuss the experiences of implementing the intervention in the study districts.

Throughout the project period, there was close collaboration between ART members and other actors, with explicit efforts by the researchers to understand the context within which the AFR approach was being implemented. The researchers attempted to capture data from the perspective of those individuals who would have to implement and sustain the AFR approach on an ongoing basis. The ART members organized sensitization workshops at the district level to generate enthusiasm for the AFR framework. Stakeholders who have been sensitized about AFR conditions include: the Regional Health Management Team, the Regional Secretariat, the District Health Forum (heads of health facilities), councillors (political leaders), the Chairperson of Health Facility Governing Committees, nongovernmental organizations (NGOs), faith-based organizations (FBOs), community-based organizations (CBOs), and heads of department.

At the request of CHMT members to have a person stationed at the district, in November 2008 the REACT project recruited a focal person who was positioned full time in the district to observe and facilitate the implementation of the project. The role of the project focal person included: documenting events related to the implementation of AFR in the district, attending the CHMT management meetings to observe the actual application of AFR in day-to-day decision-making processes, coaching CHMT members on AFR concepts and their application, and capturing the reactions of different stakeholders to the implementation of the AFR framework in the district.

### Data collection techniques

This article is based on two major sources of data: analysis of documents and key informant interviews. Documents analyzed included minutes of the ART, CHMT, and annual planning and priority-setting reports. Key informant interviews were conducted with various stakeholders in the district and region. Respondents were purposively selected based on their position in the management of district health systems. These included: the CHMT, Council Health Service Board members, local government officials, FBOs and NGOs. In addition, health workers in both public- and faith-based health facilities were selected based on the position they held at the facility. Respondents were mainly heads of the health facility or section. Furthermore, all six representatives of the marginalized groups, namely women, youth, elderly, disabled, and people living with HIV/AIDS, who joined the CHMT for priority setting and budget discussion were interviewed. An interview guide was developed and consisted of a series of questions. Respondents were also asked to identify changes in the planning and priority-setting process over the previous 2 years. Consistent with qualitative research methods, an open stance was maintained, probing into emerging themes and seeking clarification when necessary. Interviews were conducted in two phases. Twenty-one interviews were carried out with various stakeholders in the district toward the end of the REACT project in August 2010. An additional 14 interviews were carried out 1 year after the end of the project in April 2012 by the researcher (S. M.) who was not directly involved in the implementation of the project in the district. In the second phase, respondents included only those who were directly involved in the priority setting and budget discussions namely CHMT members and representatives of the communities. In total, 35 interviews were carried out and analyzed as indicated in Table 4.

**Table 4:** List of respondents

		Sex		
			
	Category of respondents	Male	Female	Total
1	Core Council Health Management Team (CHMT)	5	2	7
2	Co-opted CHMT members	3	1	4
3	Council Health Service Board (community representatives)	1	1	2
4	Public health facility workers	6	4	10
5	Faith-based health facility workers	2	0	2
6	Representatives from special groups (women, youth, elderly, disabled, and people living with HIV/AIDS)	4	2	6
7	District local government officials	2	1	3
8	Nongovernmental organization	1	0	1
9	Regional Health Management Team	2	1	3
	Total	24	11	35

### Data analysis

Thematic framework was adopted in analyzing documents and interviews. An initial code manual was developed based on the research questions and this provided a framework for the initial categorization of text. Interviews were transcribed verbatim to produce transcripts of narrative text for thematic analysis. Thematic analysis with open coding was performed, whereby each segment of interview text that related to the factors of interest was coded as a provisional theme. These codes were descriptive and linked with representative examples from the original text. Themes were identified, coded, recoded, and classified by examining regularities, convergences, and divergences in the data (23). We also highlighted differences and convergences in responses from respondents. Finally, data were summarized and synthesized, retaining as much as was possible key terms, phrases, and expressions of the respondents. To ensure the quality of results, data were triangulated to allow comparison across sources and different categories of respondents and subsequently discussed for final interpretation and presentation.

## Results

This section presents results organized in two major themes: the process of involving community representatives and perceived changes after involvement of representatives of communities in planning and priority-setting processes. Challenges of promoting community participation are also presented in tandem with the perceived changes.

### How did the process of involving community representatives unfold over time?

AFR requires the involvement of different kinds of stakeholders in the priority-setting process to include a wide range of values, enhance legitimacy, and facilitate the implementation of the decisions made. To achieve this condition, the researchers advised the CHMT to broaden stakeholder involvement with emphasis on community representation in the planning and priority-setting process. Analysis of the minutes and project implementation reports revealed that the process of involving stakeholders unfolded in stages.

Initially in 2008, the CHMT member decided they would give priority to the special groups and identified five groups in the community including: women, youths, elderly, disabled, and people living with HIV/AIDS. These groups are well-known in many districts in Tanzania and they normally have their own associations but are rarely involved in the planning and priority-setting processes. The CHMT members felt they had little knowledge of the needs of these groups. In addition, the CHMT felt that these groups face problems that are different from those experienced by other members of the community. Therefore, CHMT members realized that through these representatives it would be possible to obtain priorities that reflect the actual needs of the communities.

However, this idea did not materialize. Instead, the CHMT incorporated an NGO representative and one person from the Regional Health Management Team in the District planning team. In the second stage, in 2009, the CHMT broadened the team further by incorporating the Medical Officer in-charge (MOi/c) of the District Hospital, Matron (District hospital) and Coordinators of the programs in the district.

The CHMT put forward various reasons for dropping the idea of involvement of groups from the community in district health priority setting. First, some members of the CHMT upheld the perception that the community does not understand the disease pattern in the district and that they had little knowledge of health problems. The CHMT suggested that instead of involving community representatives in the priority-setting sessions, CHMT members should visit villages to solicit community priorities. The researchers opposed this suggestion but to sustain the action research spirit of the REACT project they agreed to the CHMT's idea of going to the villages. The CHMT were surprised how the lay people provided useful information. For instance, villagers raised issues of poor quality of healthcare delivery services in the health facilities, bad language of the health workers, general cleanliness in the health facilities, and ineffectiveness of the drugs dispensed by the facilities.

Second, the CHMT said that it had no authority to allow participation of other stakeholders in the district health priority setting. Such authority rests with the District Executive Director. The CHMT raised fear that if the names of new participants in the district health priority-setting team appear in the District Health Plan, the higher authorities would not approve the plan. Third, the CHMT argued that the district had not allocated funds to cover the costs associated with participation of additional stakeholders.

Responding to the CHMT's concerns, the researchers kept on reminding decision makers about the importance of stakeholder involvement in district-level planning and priority setting, particularly stakeholders from the ‘demand side’ including users of the health services.

After discussions during the ART meetings, the CHMT in late 2010 revived the idea of incorporating special groups from the communities. The CHMT members requested each group to nominate one person who would be invited in the district annual planning and budgeting process. The selection of the members in each group was to be based on two main criteria: community representatives should be literate (able to read and write) and they should not be occupied with other engagements that would jeopardize their participation in the district planning and priority-setting processes. Each group identified one representative and submitted the name of the nominee to the CHMT members for approval. Six community representatives were nominated in total. These community representatives were only expected to be involved in annual planning and the budgeting process, which happens once per year.

The CHMT members requested researchers to provide sensitization and training to the representatives of the identified groups to enable them to participate effectively in the priority-setting process. The one-day training covered participatory planning and priority setting as well as basic knowledge on the governance of the district health systems. Consequently, these community representatives were invited to participate during the 2011/2012 and 2012/2013 planning sessions.

### What were changes after involving community representatives in priority setting?

Analysis of interviews indicates that involvement of community representatives produced a number of results and created a number of challenges as indicated below.

#### Identification of community needs and priorities

Respondents felt that stakeholder participation in the planning and priority-setting process had increased significantly. This was also evident by the involvement of the six representatives from marginalized groups of the society: women, youth, disabled, elderly, and people living with HIV/AIDS. The vast majority of respondents had the opinion that the involvement of representatives from the community has helped improve the priority-setting process. They argued that the CHMT now gets information that they would not have obtained without the involvement of representatives of the community. One responded noted:

Before we began involving communities, there were many issues which CHMT members did not think that they were priorities of the communities. After we started involving representatives from communities, they have been able to identify a number of important issues. (interview with a CHMT member)

Similarly, respondents from the faith-based and NGOs category had the opinion that widening of stakeholder involvement has helped to bring different views and perspectives to the planning and priority-setting process.

However, the respondents identified a number of challenges. First, while the CHMT members have started involving special groups from the community in the priority-setting process, there is no financial support provided to the representatives of the communities to enable them to perform their roles effectively. Second, almost all community representatives reported that the duration of 1 day for the pre-planning meeting was too short and that not enough time was given to discuss priorities presented by different groups.

Third, the general feeling among community representatives was that the CHMT members provided short notice to community representatives about planning and priority-setting meetings, usually 1 day before the meeting. Some respondents illustrated this predicament as follows:

Even though we have been participating in the planning meetings, we need adequate preparation. Most often, we receive short notice for the meeting. For example, in the last planning meeting I got information about the meeting a day before. (IDI with a representative of community)

Another respondent added:

The main problem is that the current system used by the district is not good. We were supposed to get invitation at least a week before the meeting in order to enable us elicit views from our members. I think communication is not effective. (IDI with a community representative)

#### Perceptions of transparency and accountability in the priority-setting process

In general, the majority of respondents felt that the district health officials were increasingly developing a culture of openness. They became more transparent by publicizing district health priorities to different stakeholders. One respondent remarked:

Transparency has increased profoundly over the past two years. Nowadays, even priorities and budgets are posted on notice boards for people to see them. (IDI with a community representative)

This was echoed by the respondents from the FBO, NGO, as well as the regional level. Respondents had the feeling that there had been an increased openness and participation in the planning and priority-setting process. When asked what changes, if any, they had noticed in the priority setting, one respondent remarked:

Certainly, Mbarali has used the REACT project very well. I myself have participated in some district health board meetings. I have seen that there is more transparency in Mbarali compared to other districts. (IDI with a regional health officer)

It was evident that publicizing district health priorities was a notable change in the management culture of the CHMT. The analysis of interviews and minutes of the ART indicated that although the mechanisms for publicity were *ad hoc*, various stakeholders were impressed with the decision to make the priorities known to them.

Some local government officials and regional health officers attributed the increased transparency to the new district health leadership. They pointed out that in the past health, workers were divided into antagonistic groups and that most of the decisions were made by only a few management leaders. The new leadership was reported to be more transparent and one that invited divergent views in the meetings.

However, a few representatives of the community reported that once the process of developing the district health plan and budget has been completed and approved, the CHMT did not provide feedback on the approved priorities. Also, the CHMT members did not report implementation status of the approved priorities. In addition, almost all Council Health Service Board members pointed out that they had not been involved in preparing district health plans and budgets. One respondent expressed:

There is no room for us to give our opinions. District plans are read to us just for information. The secretary of the board tells us how far we have gone. We do not give our opinions. We just accept what has been written by the experts. (IDI with a CHSB member)

### Perceived improvement of quality of care

Almost all respondents felt that health services in a number of areas have improved over the past 2 years. These include: improvement of district hospital services; Care and Treatment Centres for people living with HIV/AIDS; and the distribution of condoms to prevent the spread of HIV and sexual transmitted infections (STIs).

With respect to the district hospital, a number of examples were provided by the respondents. First, it was indicated that at the district hospital elders have their own doctor and get special treatment in the wards as well as other outpatient departments. One respondent said:

During the planning meeting of 2011/2012 we requested to get our doctor so that to shorten waiting time at the district hospital. We have special room and doctor who is taking care of elderly group only. (IDI with a community representative)

Second, the vast majority of respondents reported changes in the use of language by health workers. The respondents reported that in the past, many health workers at the district hospital used to abuse patients. Almost all respondent said that health workers have significantly changed their behavior and attitudes toward patients. One respondent expressed:

There are significant changes. For example, in the past pregnant mothers were not treated with respect. However, after we presented these during the planning and priority-setting meetings, we have witnessed changes. The abusive language by the health workers has largely vanished. (IDI with a community representative)

Third, almost all respondents reported an improvement in the renovation of infrastructures at the district hospital to provide easy access to physically handicapped individuals. The disabled had complained that infrastructures at the district hospital were not user friendly. In particular, at the district hospital there were stairs which made it difficult for them to access the service areas of the hospital. They suggested that these stairs were removed. The CHMT agreed with their suggestion and the stairs were removed. During the field visit, the researchers witnessed a number of renovations at the district hospital to enable easy access to services for the physically handicapped group of the society.

As for improvement of care for HIV/AIDS patients, almost all respondents from the CHMT and community representative categories reported that people living with HIV/AIDS had complained that there were long waiting times when they visited the district hospital. They also complained about frequent changes of drug regimes for people living with HIV/AIDS. Before that the district used to provide Antiretroviral Therapy and other related drugs for people living with HIV/AIDS only 2 days per week. Because of the suggestions and demand from the community representatives, the CHMT members decided to add 2 more days for providing services to HIV/AIDS patients. In addition, the CHMT introduced mobile Care and Treatment Clinics to meet the increased demand for antiretroviral services.

Furthermore, the vast majority of respondents reported an improvement in the distribution of condoms to prevent the spread of the HIV/AIDS and STIs. Traditionally, the CHMT distributed free condoms to the health facilities only. The youth representative had suggested that condoms should be made available in the entertainment venues such as bars and guest houses instead of being distributed at the health facility. This would enable young people to have access to the condoms. Based on the suggestions by the youth representatives, the CHMT members started supplying condoms since 2010 to bars and guest houses, for young people to access easily.

Analysis of interviews from different categories of respondents indicated that trust between the communities and health workers has improved. As for the reasons for improved trust, respondents had different views. The majority of health workers and CHMT members attributed changes of trust to the involvement of community representatives and increased openness in the priority-setting process. One respondent exemplified this way:

Involvement of representatives of different groups in the society has largely improved our relationship with the community. I think this has also improved trust between health workers and patients. (IDI with a health worker)

Other respondents attributed increased trust to the improvement of quality of health services, in particular, the availability of drugs and medical supplies. Furthermore, some local government officials attributed changes of trust to the new district health management and increased transparency of the district health managers. However, one respondent reported that there had been no changes in trust. The respondents pointed out that patient had very little trust regarding the health workers.

## Discussion

This article aimed to illuminate the experience of promoting community participation in priority setting through the Response to Accountable priority setting for Trust in health systems project in Tanzania. The findings show that the process of involving representatives of special groups in the preparation of the district health plans and budgets took a long time. This is because initially, the CHMT members did not see the importance of engaging the public in priority setting. This manifested itself in their perception that the community representatives do not understand the disease pattern in the district and that they had little knowledge of health problems. This is not surprising because traditionally, in Tanzania and elsewhere, this myth had been accepted that health is something medical professionals are responsible for, they are providers, and communities are service users. However, after inputs on district health plans and priorities, as a result of the involvement of community representatives, the CHMT members came to learn that communities are important in the priority-setting process.

The findings also showed the importance of external facilitation and support in enabling health professionals and community representatives to arrive at effective working arrangement. It is evident from the study that despite the existence of guidelines for community participation, it took a long time for the CHMT members to appreciate the value of community participation in the priority-setting process. The researchers’ role in promoting community participation in the study setting cannot be underestimated. Apart from pushing for the community participation, researchers trained representatives of the community groups on planning and priority-setting processes. Similar experiences have been reported elsewhere. For example, Paxman and colleagues (24) documented how training and support provided by NGO to 620 village health committees in India helped to improve reproductive and child healthcare coverage and outcomes. In addition, Bjorkman & Svensson (25) reported an intervention in Uganda that included structured and facilitated interactions among health facility committees, communities and health facility staff to develop an effective monitoring and a health information system.

Another reason for the need of an external facilitator is that the question of community participation speaks to the power relations and community capacity issues. Evidence shows that power is centralized despite the existence of impressive structures for community participation (15, 16, 26, 27). In addition, there is a lack of information about policies, laws, people's rights, and even what is happening in the country (27). Consequently, health facility committees, boards, and the public, seem unable to influence quality of the decentralized healthcare planning and priority-setting processes. In these contexts, external support is indispensable in improving community awareness of their roles and responsibilities in the district development process and broadening their understanding of the laws, regulations, and guidelines that govern district health systems.

Respondents reported a number of improvements in priority setting and service delivery. While the majority of the respondents attributed these changes to the involvement of the community representatives, some factors other than community representatives might have contributed to these improvements. For example, recently the national policy environment, political leaders, and other stakeholders in the country have been vigorously pushing for an improvement in health services for the marginalized groups of society including women, disabled, elderly, and people living with HIV/AIDS. Notwithstanding the favorable national policy environment, the findings suggest that it was not until community representatives were involved in the priority setting that the outcomes being reported here were realized. The community representatives were energetically able to raise these problems during the planning meetings.

There is some evidence indicating that community participation is an important element in strengthening the health system and improving health service delivery. A Canadian study provided insights into the nature, extent and impact of citizen participation on policy and service outputs in 17 community health centers across the country. The findings from the study show that the citizens who were participants in decision-making processes of the organizations, felt that their participation ‘led to improved programs and services, and that the range of programs and services met the needs of the community’ (28). Furthermore, community health centers were seen as ‘organizations that increased community capacity through helping communities and individuals to raise awareness about health and social issues, identify community strengths and weaknesses, build shared community values, increase community and individual confidence to participate, and increase levels of trust within the community’ (28). Similarly, recent reviews in low- and middle-income countries concluded that community participation can be effective in terms of improving the quality and coverage of healthcare, as well as impacting on health outcomes (29, 30).

However, while recognizing the importance of working with community representatives, it is also imperative to note challenges embedded in the community participatory structures. Despite good roles played by community representatives in the planning and priority-setting process, it should not be taken for granted that community representatives do a good job of representing their communities. Our study has not been able to verify processes through which community representatives were selected. In addition, further research is needed to explore the extent to which community representatives represent community views and how feedback from the planning and priority-setting meetings are shared with the wider communities.

However, it is also imperative to enhance the individual capacity of service providers, administrators and elected officials to be receptive to other stakeholders, and to carry out consultative and inclusive planning processes. Effective implementation of decentralization requires strengthening both ‘supply’ and ‘demand’ sides (31). Flores and colleagues (32) working in Guatemala, have reported considerable success in transforming the attitudes of healthcare workers and officials through addressing community capacity to act as empowered citizens. The approach indicated is not a top-down change in policy or creation of more official structures, but a bottom-up addressing of how communities engage with health services on their own terms.

Furthermore, it is imperative to note that the process of promoting community participation in the district health planning and priority setting was not smooth. The traditional cultural contexts within which the project was implemented created challenges to both researchers and decision makers, which consequently slowed down the implementation of the REACT project (33). For example, the process of involving representatives of special groups in the preparation of the district health plans and budgets took a long time. This is because of the embedded tradition of health professionals’ unwillingness to involve the communities in priority settings. In addition, the CHMT members felt that they had no authority to allow participation of other stakeholders in the district health priority setting. Frequent meetings between the researchers and district health decision makers seemed to have increased the level of trust and receptivity to the adoption and implementation of the AFR innovation. In addition to formal collaborations, informal networks in the form of friendly relationships among researchers and decision makers were also imperative. Furthermore, the presence of a project focal person dedicated to the implementation of the intervention became evident in this study. In Mbarali district, the project focal person who was stationed full time in the district became part of the implementation team, and was perceived as an expert in the AFR approach to priority setting.

Methodologically, this study relied primarily on the review of project implementation documents and key informant interviews with the community representatives and district health managers who were involved in the implementation of the REACT project in the district. It is possible that participants were shaped by social desirability bias and they might have told the researchers what the latter wanted to hear. However, to minimize this risk, interviews were conducted by a researcher (S. M.) who was not directly involved in the Action Research process in the district. In addition, the study did not manage to validate statements provided by the respondents with hard data from the communities and health workers who were not directly involved in the implementation of the project. Equally important, the study did not collect hard data on the changes in health service utilization and spending patterns in the district health plans. Further research is needed to gain more insights from the perspectives of the service users. However, the study sheds light on how community participation can be promoted in the context of resource-poor settings, weak organizations, and fragile democratic institutions. Therefore, this study could help healthcare analysts, decision makers, and others improve their understanding of the role of communities in the governance of district health systems and form the foundation for many of the ongoing efforts to promote community participation in planning and priority-setting processes.

## Conclusion

It is concluded in this article that community participation in priority setting in developing countries, characterized by weak democratic institutions and low public awareness, requires effective mobilization of both communities and health systems and adequate continued procedural guidance in written form and by conducive managerial functions. Simply establishing institutional arrangements of participatory planning, priority setting, and governance in the absence of strong capacity building and support will not result in greater responsiveness to community needs and priorities. In addition, this study confirms that community participation is an important element in improving health service. Community representatives have real-life experience as users of the healthcare system and other public services and can offer insight into the values and beliefs of the public at large.
